# Commentary: Synthetic Addiction Extends the Productive Life Time of Engineered *Escherichia coli* Populations

**DOI:** 10.3389/fbioe.2018.00077

**Published:** 2018-06-12

**Authors:** Chiara Enrico Bena, Alice Grob, Mark Isalan, Carla Bosia, Francesca Ceroni

**Affiliations:** ^1^Department of Applied Science and Technology, Politecnico di Torino, Torino, Italy; ^2^Italian Institute for Genomic Medicine, Torino, Italy; ^3^Department of Life Sciences, Imperial College London, London, United Kingdom; ^4^Imperial College Centre for Synthetic Biology, Imperial College London, London, United Kingdom; ^5^Department of Chemical Engineering, Imperial College London, London, United Kingdom

**Keywords:** bioproduction, synthetic biology, metabolic burden, product-addiction, synthetic devices

Bioproduction is the process of producing added-value chemicals on large-scale using cells as biological factories. Cellular burden represents a significant problem in the scaling of fermentation processes from proof-of-concept to long-term cultures, as the load of heterologous gene expression and depletion of the cell intracellular resources cause unpredictable cellular physiological changes that can lead to decreased growth and lower production yields (Borkowski et al., 2016; Liu et al., 2018). One possible cause of the observed decreased bioproduct recovery in many bioprocessing applications is the accumulation of mutations in the employed genetic program. These mutations often lead to loss of production and rise of non-producing populations that grow better and easily overtake the growth of producing cells (Rugbjerg et al., 2018b).

In a recent paper in PNAS, Rugbjerg et al. (2018b) developed a strategy to limit the enrichment of non-producing cell populations in bioproduction-employed cell cultures by placing the genes for key growth intermediates under the control of a promoter responsive to the bioproduct being made. This strategy known as product addiction was tested in *E. coli* engineered to produce mevalonic acid in long-term cultivations (Figure 1).

**Figure 1:**
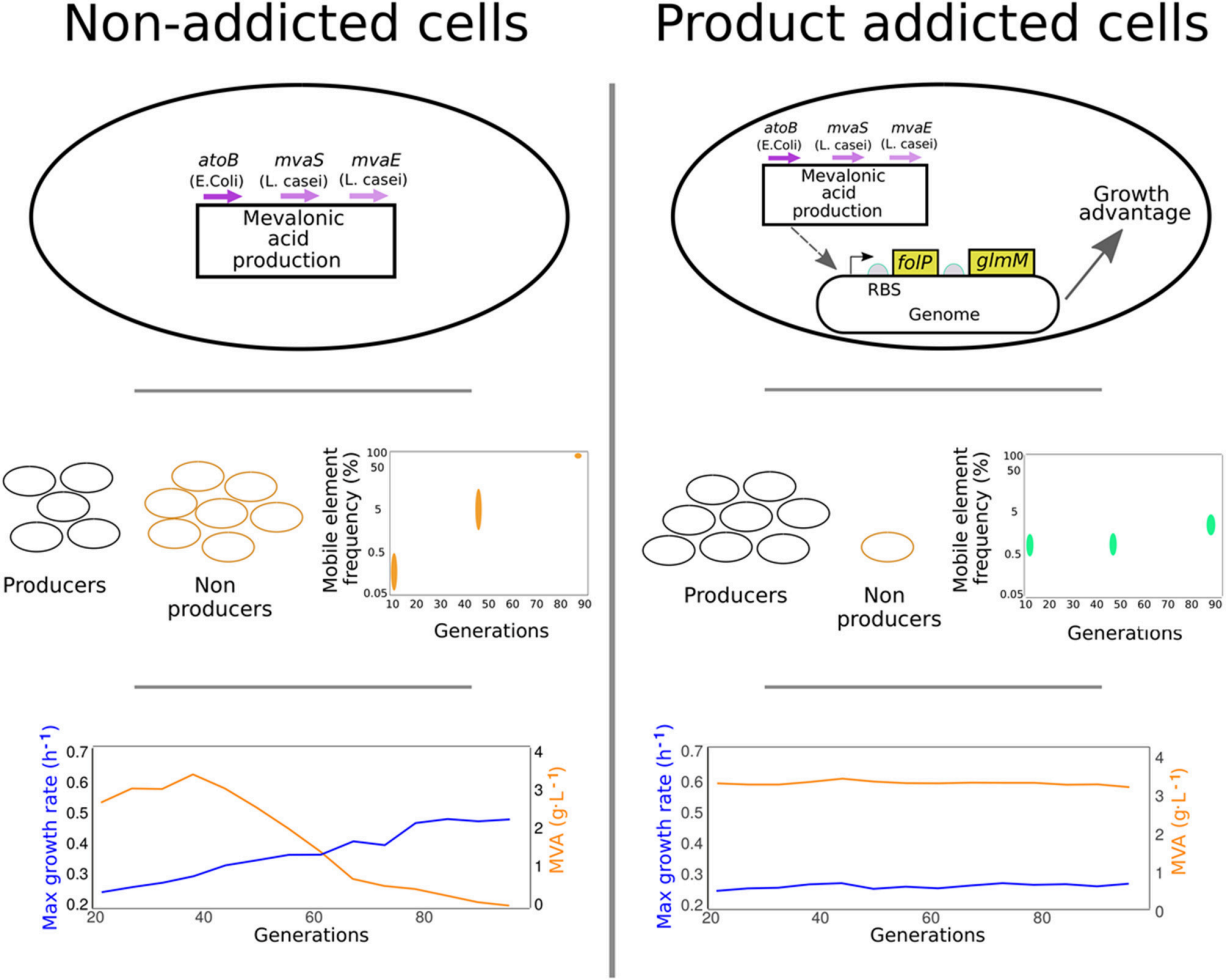
*Product-addiction*, a strategy to limit the enrichment of non-producing cell populations in bioproduction-employed cell cultures is described by Rugbjerg et al. *E. coli* cells engineered to produce mevalonic acid in long-term cultivations are used to demonstrate this concept. When cells producing mevalonic acid are grown over a longer cultivation period, subpopulations of non-producing cells arise that overtake the growth of producing cells. These cells are shown to carry mobile elements from the bacterial genome inserted in the DNA construct that lead to lower production and better growth (Left). In product addicted cells, authors place genes essential for growth (*folP* and *glmM*) under the control of a promoter that is responsive to the target product, mevalonic acid. Growth of non-producing cells is greatly diminished and cells maintain robust production of mevalonic acid over time.

To achieve *product addiction*, the authors selected the chromosomal genes *glmM* and *folP* for a positive feedback control associated with the product. Both of these genes are known to supply essential intermediates for cell growth. In initial screening, the *folP-glmM* operon was placed under the control of the P_BAD_ arabinose-inducible promoter and integrated into the genome. To avoid imposing extra-burden to cells, different library variants were designed where promoter and RBS strength were tuned. By analyzing the growth profiles in the presence and absence of the arabinose inducer, the authors identified a good genetic design that matches the requirements for both responsive-growth regulation and low burden. In order to make product addiction specific to mevalonic acid, the inducible promoter guiding the expression of the operon was then replaced by the AraC_mev_ promoter, known to be responsive to the mevalonic acid product.

The authors then went on to compare growth profiles and product yields of mevalonic acid in both addicted and normal *E. coli* cells, in presence of a mevalonic acid-producing construct. During long-term growth in bioreactor conditions, the addicted population grew slower than the non-addicted one, but was able to maintain constant and robust mevalonic acid production over time, thus yielding higher titers overall. The addicted population also did not display any outbreak of non-producing subpopulations, as seen with non-addicted cells. These populations were also examined using deep-sequencing techniques, allowing the authors to investigate the presence of genetic heterogeneity in both addicted and non-addicted cells. Interestingly, the sequencing results for the mevalonic acid-producing pathway identified mobile insertion elements from the native host genome as the main cause for the disruption of the genetic program in non-producing cells. This confirmed work released in parallel by the same group investigating the mutation and failure modes of constructs in *E. coli* (Rugbjerg et al., 2018a).

This elegant piece of research by Rugbjerg et al. advances metabolic engineering of bacterial cells, by building on previous achievements in synthetic biology and engineered feedback control. In the last decade, bacterial synthetic biology has witnessed the development of a plethora of synthetic systems and tools for the rational control of gene expression (Bradley et al., 2016; Nielsen et al., 2016). Together with our ability to engineer novel genetic devices, our ability to use them for sensing and responding to external and intracellular stimuli has also improved, leading to advances in applications where substrate sensing is important, such as in metabolic engineering.

The approach of Rugbjerg et al. makes use of insights from the synthetic biology field relating to circuit engineering and burden analysis, and combines them for a metabolic engineering application. Previous approaches exploited well-controlled bio-reactor conditions or adaptive evolution to improve synthesis of growth-associated products, leveraging natural selection for reducing metabolic burden (Wu et al., 2016). More recently, Dahl et al. made use of intracellular biosensors to detect the level of a metabolic intermediate and control enzyme expression levels to maximize the product (Dahl et al., 2013). Overall these systems remain costly as they make use of expensive inducers or antibiotics for selection.

By contrast, Rugbjerg et al. smartly exploit non-conditional dependency of growth on the fermentation product so as to render production independent from external inputs. They take a rational approach in circuit design to identify the optimal *folP-glmM* operon variant for the sustained growth of addicted-cells while conferring tunable response to variant product concentrations.

The general strategy also proves effective as a way to reward producing cells at the expense of non-producing ones limiting the detrimental effects of burden on the population homogeneity. The emergence of escape mutants as a consequence of cellular burden was previously shown by Sleight et al. (Sleight and Sauro, 2013). Long-term batch growth of producing cells was also reported to be subject to construct inactivation by random insertion of transposable elements from the host genome (Ceroni et al., 2015). Rugbjerg et al. overcome this problem by conferring a growth advantage to producing cells that display more robust production of mevalonic acid over time.

This paper from Rugbjerg et al. represents a fine example of how advances in burden characterization and genetic system design can be exploited to significantly improve yields in bioproduction processes. The strategy they develop is autonomous from external stimuli and allows maximization of product recovery as a consequence of more robust gene expression over time. A potential drawback of the approach presented is its non-universality: *Product addiction* is achieved by exploiting a specific promoter responsive to mevalonic acid. If another product were being made instead, a different sensor promoter would need to be identified and the build-and-test cycle of the system repeated *ad hoc*. Ideally, in the future, variant but related strategies will be developed that are also universal and portable. Overall, the work by Rugbjerg et al. proves the power of the synthetic biology approach toward the optimization of bacterial metabolic engineering.

## Author contributions

All authors listed have made a substantial, direct, and intellectual contribution to the work, and approved it for publication.

### Conflict of interest statement

The authors declare that the research was conducted in the absence of any commercial or financial relationships that could be construed as a potential conflict of interest.
